# Effect of unplanned conversion to open surgery on resection margins and complications in laparoscopic pancreaticoduodenectomy: a systematic review and meta-analysis with meta-regression

**DOI:** 10.1308/rcsann.2025.0078

**Published:** 2025-10-01

**Authors:** S Hajibandeh, S Hajibandeh, J Alazab, H Alazab, M Safiru, T Satyadas

**Affiliations:** ^1^ Swansea University, UK; ^2^ Morriston Hospital, UK; ^3^ Manchester Royal Infirmary Hospital, UK; ^4^ Queen Elizabeth Hospital, UK; ^5^ Ministry of Health, Jordan

**Keywords:** Pancreaticoduodenectomy, Laparoscopy, Conversion to open, Mortality, Morbidity

## Abstract

**Introduction:**

We aimed to investigate the effect of unplanned conversion to open surgery during laparoscopic pancreaticoduodenectomy on resection margins and complications.

**Methods:**

A systematic review and meta-analysis (proportion and comparison models) with meta-regression using random-effects modelling compliant with PRISMA statement standards was conducted. All studies with a minimum sample size of 15 patients reporting conversion to open surgery in patients undergoing laparoscopic pancreaticoduodenectomy were included. The outcomes included R0 resection, Clavien-Dindo ≥3 complications, and 30-day mortality.

**Findings:**

A total of 44 studies comprising 6,108 patients were included. Conversion occurred in 11.3% (95% confidence interval (CI) 9.1–13.5). The reason for conversion was bleeding in 27.9% (16.3–39.5%), technical difficulties in 46.5% (95% CI 33.7–59.4), oncological concerns in 29.2% (95% CI 18.2–40.2) and iatrogenic injuries in 7.7% (95% CI 3.4–12.1). Multivariable meta-regression analysis showed that conversion did not affect R0 resection (coefficient: −0.228, *p*=0.307), Clavien-Dindo ≥3 complications (coefficient: 0.129, *p*=0.609) and 30-day mortality (coefficient: −0.013, *p*=0.647). The outcomes were not affected by the reasons for conversion. Comparison meta-analysis showed that conversion does not affect R0 resection (risk difference (RD): −0.07, 95% CI −0.17–0.03, *p*=0.18), Clavien-Dindo ≥3 complications (odds ratio: 2.17, 95% CI 0.67–6.99, *p*=0.20) and 30-day mortality (RD: 0.02, 95% CI −0.04–0.07, *p*=0.57).

**Conclusions:**

Unplanned conversion to open surgery, regardless of the reason for conversion, may not affect resection margins and complications in laparoscopic pancreaticoduodenectomy (moderate certainty). Conversion during laparoscopic pancreaticoduodenectomy should not be seen as a failure because it has no negative impact on outcomes; however, not converting when indicated will undoubtedly have negative impact.

## Introduction

Pancreaticoduodenectomy remains a complex and challenging procedure; however, improvements in perioperative care and advances in operative techniques have expanded the indications for minimally invasive pancreatic surgery.^1–4^ It has been shown that laparoscopic pancreaticoduodenectomy performed by an experienced surgeon does not compromise oncological outcomes but may reduce postoperative morbidity and length of hospital stay.^5^ However, retroperitoneal dissection around major vessels and need for constructing complex anastomoses make laparoscopic pancreaticoduodenectomy a technically difficult and time-consuming procedure with a risk of injuring major vessels that can lead to life-threatening complications.^6^

Laparoscopic pancreaticoduodenectomy may require unplanned conversion to open surgery due to bleeding, technical difficulties, oncological concerns or iatrogenic injuries. The risk of conversion to open surgery may range between 3.1 and 24.1%.^5^ The prognostic significance of unplanned conversion to open surgery during laparoscopic pancreaticoduodenectomy and its impact on resection margins, postoperative morbidity and mortality remain poorly understood. Consequently, we aimed to conduct a comprehensive meta-analysis with meta-regression to investigate the prognostic significance of conversion to open surgery during laparoscopic pancreaticoduodenectomy.

## Methods

### Methodological and reporting compliance

The methodology of the study followed the Cochrane Handbook for Systematic Reviews (version 6.4) and the reporting of the study followed the Preferred Reporting Items for Systematic reviews and Meta-Analyses (PRISMA) 2020 statement standards.^7,8^

### Eligibility criteria

#### Study design

All randomised controlled trials, cohort studies and case series with a minimum sample size of 15 patients were considered eligible for inclusion in this study. Considering that conversion to open surgery cannot be randomised due to ethical reasons, conducting a randomised controlled trial in this setting is not feasible; however, if a randomised controlled trial had a laparoscopic arm, the laparoscopic arm was considered for inclusion. Moreover, if an observational study had multiple arms, only the laparoscopic arm(s) was eligible for inclusion. Meta-analyses, systematic reviews, review articles, scoping reviews, case reports and correspondence articles were excluded.

#### Population

All patients aged 18 years or over who underwent laparoscopic pancreaticoduodenectomy for malignant or benign indications were eligible.

#### Prognostic factor

Conversion to open surgery due to any reason was considered as prognostic factor of interest. The reasons for conversion were classed as bleeding, technical difficulties, oncological concern and iatrogenic techniques. The reason for conversion was determined based on intraoperative indication for conversion as reported in the included studies.

#### Outcomes

Achievement of R0 resection, Clavien-Dindo ≥3 complications and 30-day mortality were the outcome measures.

Based on the above criteria, a study was only defined as eligible if it reported risk of conversion to open in at least 15 adult patients undergoing laparoscopic pancreaticoduodenectomy.

### Information sources and search strategy

Two independent authors used search keywords, limits, thesaurus headings and operators to develop a search strategy which was used in the following electronic sources: MEDLINE, Scopus, CENTRAL, the ISRCTN registry, the ICTRP registry and ClinicalTrials.gov. The following combination of keywords was used: (pancreaticoduodenectomy [MeSH Terms] OR pancreaticoduodenectomy) AND (laparoscopic OR laparoscopy [MeSH Terms]). The search had no language restrictions and the last date for the search was 10 April 2025. The reference lists of the included studies were also searched for more eligible studies.

### Study selection, data collection and data items

The titles and abstracts of the identified articles were reviewed by two separate authors, and the full text of potentially eligible articles was retrieved to select the eligible articles. A third author was consulted if there was disagreement in study selection between the first two authors. The data items (bibliometric parameters, study design, characteristics of included population, follow-up duration, age, male sex, body mass index, malignant indication for operation, conversion to open, reason for conversion, R0 resection, Clavien-Dindo ≥3 complications and 30-day mortality) were determined by the authors at the protocol development stage followed by a pilot-testing technique of randomly selected studies. Data were collected by two independent authors.

### Study risk of bias and evidence certainty assessment

The risk of bias in the included studies was evaluated by two separate authors using the QUIPS (Quality In Prognosis Studies) tool. The certainty of evidence was evaluated using the GRADE system.^9^ If there was any discrepancy in assessment by the first two authors, a third author provided opinion. An independent author was consulted if there was any disagreement.

### Effect measures and synthesis methods

OpenMeta[Analyst] software was used for proportion meta-analysis and meta-regression; RevMan Web was used for comparison meta-analysis. The DerSimonian-Laird random-effects method (confidence level: 95%; correction factor: 0.5) was used in the proportion meta-analysis model to calculate the weighted pooled risks of conversion to open surgery, R0 resection, Clavien-Dindo ≥3 complications and 30-day mortality. A multivariable meta-regression analysis including conversion to open, age, body mass index and malignancy as independent variables was modelled to examine the effect of conversion and reason for conversion on R0 resection, Clavien-Dindo ≥3 complications and 30-day mortality. In the comparison meta-analysis model, the risks of R0 resection, Clavien-Dindo ≥3 complications and 30-day mortality were compared between patients with open conversion and those without conversion. Odds ratio (OR) or risk difference (RD) were used as summary measures as appropriate. When there was no event in both groups in more than 20% of the studies, RD was used instead of OR. The unit of analysis was individual patient and intention-to-treat data were used for the analyses. Statistical heterogeneity was measured as *I*^2^ using Cochran’s Q test (*χ*^2^), and heterogeneity was classified as low when *I*^2^ was 0–25%, moderate when *I*^2^ was 25–75% and high when *I*^2^ was 75–100%. Separate analysis for studies with low overall risk of bias and leave-one-out analysis were performed as sensitivity analyses.

## Results

### Study selection and study characteristics

The search yielded 1,383 articles. Screening the titles and abstracts directly excluded 1,325 articles because they did not meet principal eligibility criteria. The full-text review of the remaining 58 articles also excluded 14 articles (ten studies did not report conversion rate; four studies combined all minimally invasive approaches without stratified data on laparoscopic cases). Consequently, 44 studies comprising 6,108 patients were included.^10–53^ The study PRISMA flow diagram is shown in Supplementary Figure 1. The baseline characteristics of the included studies and included populations are shown in Supplementary Table 1 and Supplementary Table 2, respectively.

### Risk of bias in studies

Based on the QUIPS tool, the risk of bias in study participation was low in all studies; the risk of bias in study attrition was low in all studies; the risk of bias in prognostic factor measurement was low in all studies; the risk of bias in outcome measurement was low in all studies; the risk of bias due to study confounding was low in 35 studies and unclear in 9 studies; the risk of bias in statistical analysis was low in all studies (Supplementary Table 3).

### Conversion to open and reasons for conversion to open

**Figure 1:**
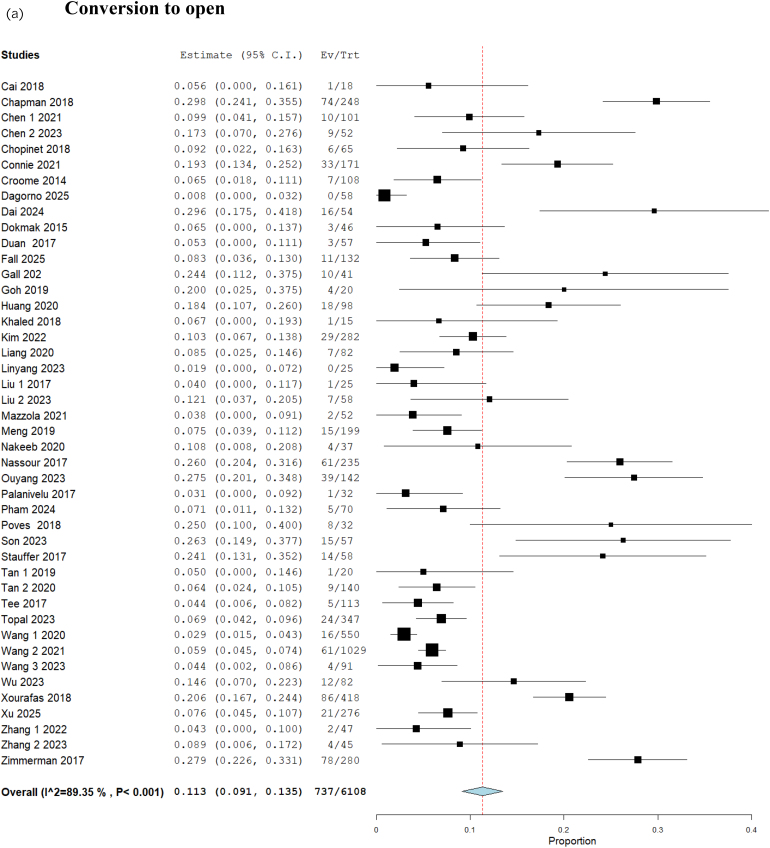

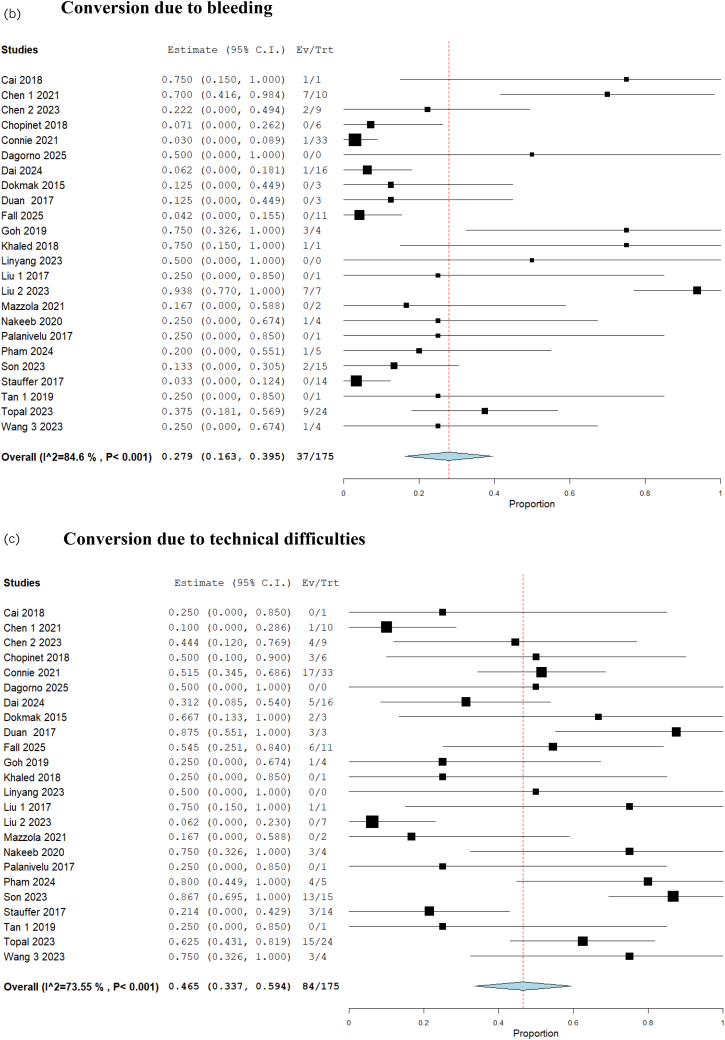

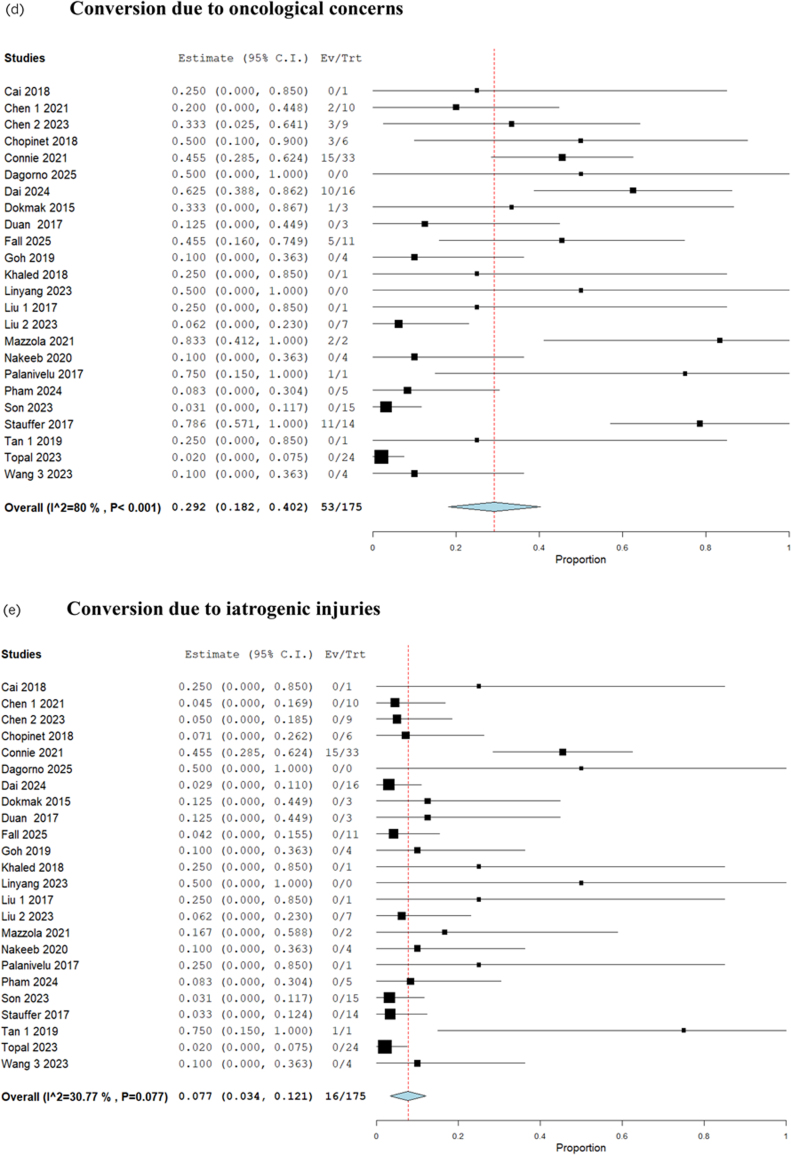
Forest plots for proportion meta-analysis of (a) conversion to open, (b) conversion due to bleeding, (c) conversion due to technical difficulties, (d) conversion due to oncological concerns and (e) conversion due to iatrogenic injuries.

Analysis of 6,108 patients from 44 studies showed that conversion to open surgery occurred in 11.3% (95% confidence interval (CI) 9.1–13.5; 737/6,108). The statistical heterogeneity was high (*I*^2^=89%) and the GRADE certainty was moderate (Figure 1a). The reason for conversion was bleeding in 27.9% (16.3–39.5%) (Figure 1b), technical difficulties in 46.5% (95% CI 33.7–59.4) (Figure 1c), oncological concerns in 29.2% (95% CI 18.2–40.2) (Figure 1d) and iatrogenic injuries in 7.7% (95% CI 3.4–12.1) (Figure 1e).

### Conversion to open and R0 resection

**Figure 2:**
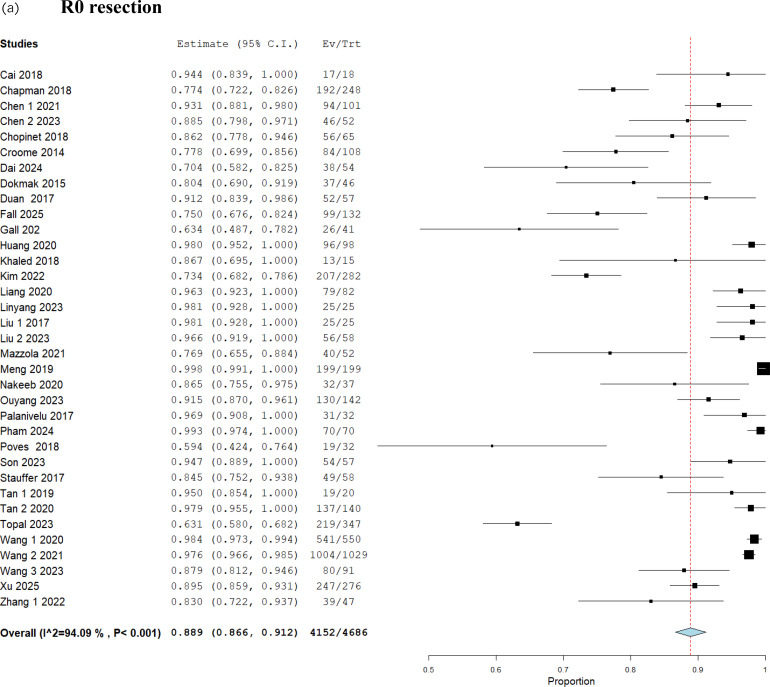

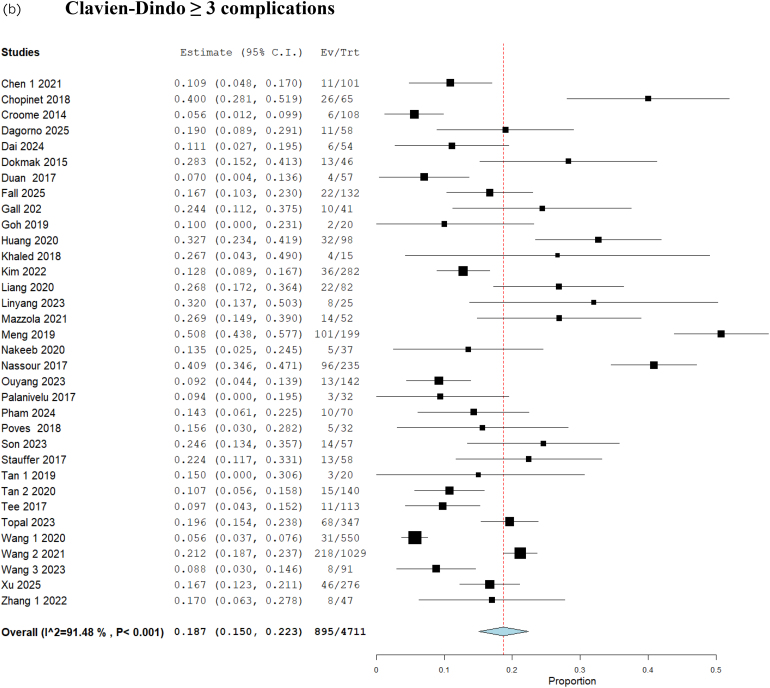

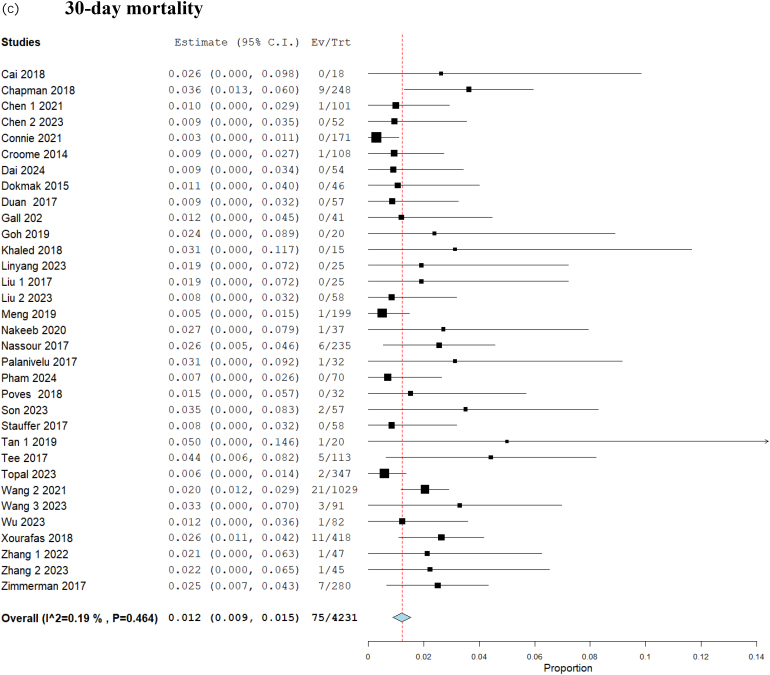
Forest plots for proportion meta-analysis of (a) R0 resection, (b) Clavien-Dindo ≥3 complications and (c) 30-day mortality.

**Figure 3:**
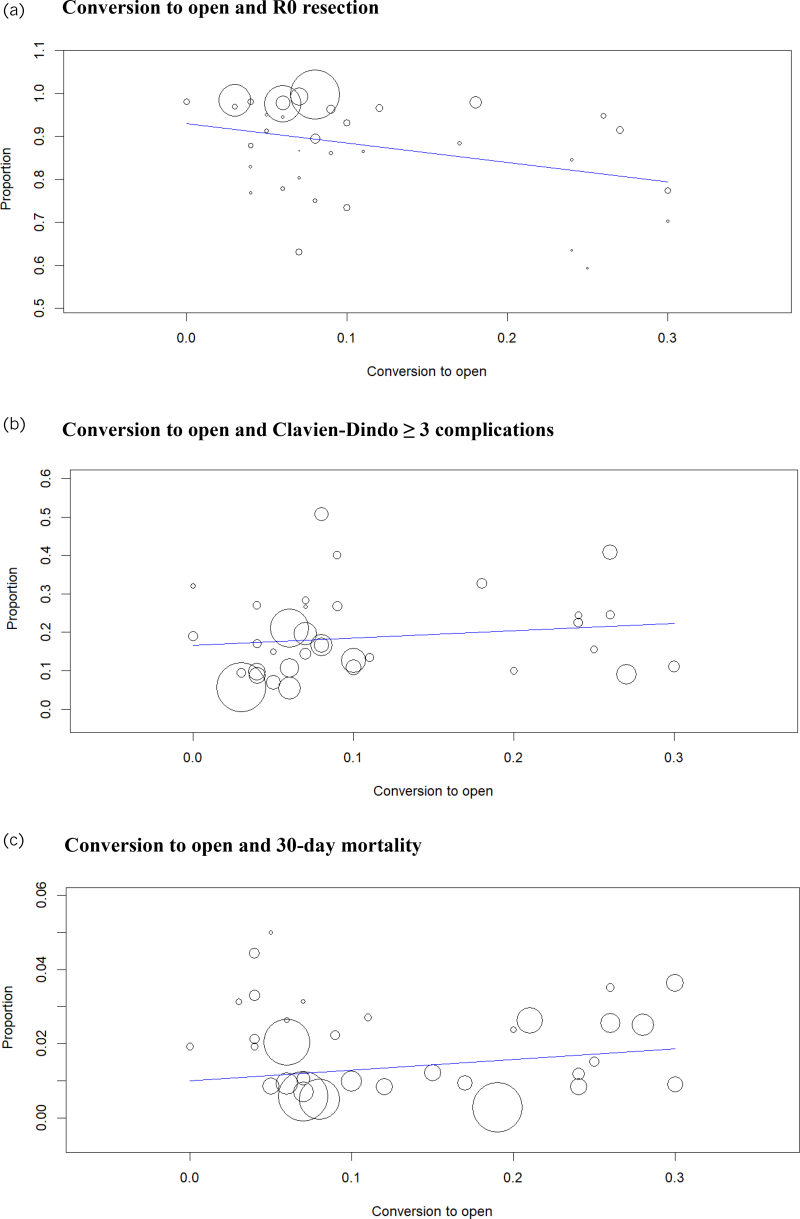
Bubble plots for meta-regression analysis for (a) conversion to open and R0 resection, (b) conversion to open and Clavien-Dindo ≥3 complications and (c) conversion to open and 30-day mortality.

**Figure 4:**
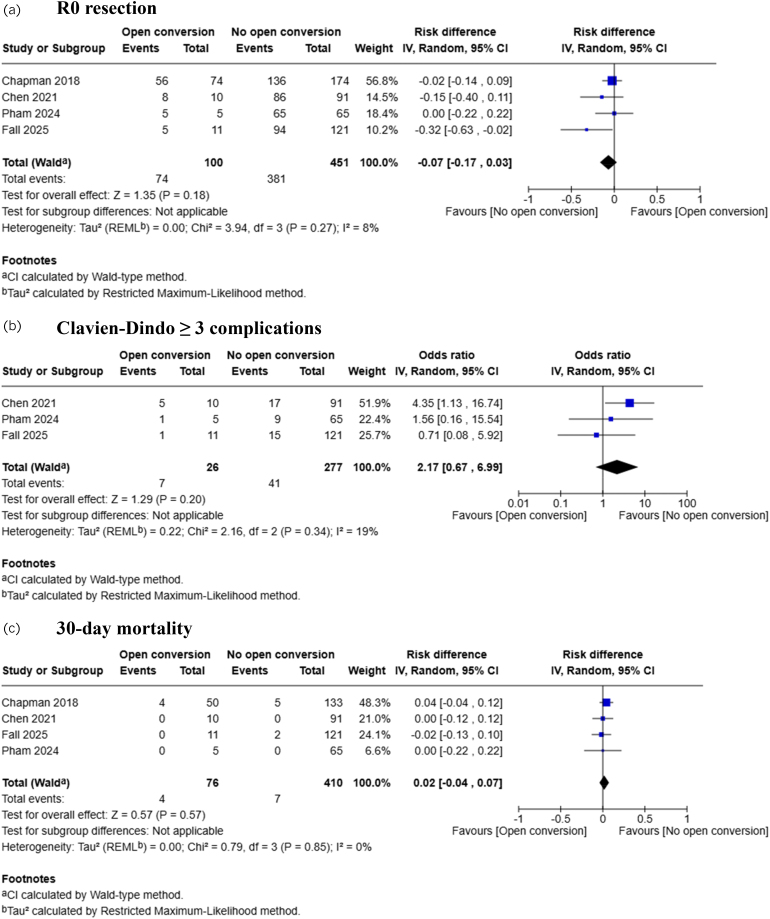
Forest plots for comparison meta-analysis of (a) R0 resection, (b) Clavien-Dindo ≥3 complications and (c) 30-day mortality.

Analysis of 4,686 patients from 35 studies showed that R0 resection was achieved in 88.9% (95% CI 86.6–91.2) (Figure 2a). Multivariable meta-regression analysis showed that conversion to open did not affect R0 resection (coefficient: −0.228, *p*=0.307) (Table 1, Figure 3a). Subgroup analysis based on reasons for conversion showed that R0 resection was not affected by conversion due to bleeding (coefficient: 0.051, *p*=0.427), conversion due to technical difficulties (coefficient: –0.005, *p*=0.938), conversion due to oncological concerns (coefficient: −0.078, *p*=0.228) and conversion due to iatrogenic injuries (coefficient: 0.077, *p*=0.466) (Table 2). Comparative meta-analysis of 551 patients from four studies showed no difference in R0 resection between patients with and without open conversion (RD: −0.07, 95% CI −0.17–0.03, *p*=0.18) (Figure 4a). The GRADE certainty was moderate.

**Table 1:** Results of multivariable meta-regression analysis for the outcomes

	Dependent variables					
Independent variables	R0 resection		Complications		30-day mortality	
	Coefficient	*p*-value	Coefficient	*p*-value	Coefficient	*p*-value
Conversion to open	−0.228	0.307	0.129	0.609	−0.013	0.647
Age	−0.009	0.055	−0.004	0.335	−0.001	0.07
BMI	−0.014	0.184	0.012	0.374	0.003	0.035
Malignancy	−0.069	0.453	0.003	0.978	0.014	0.381

BMI = body mass index.

**Table 2:** Results of multivariable meta-regression analysis based on reasons for conversion to open surgery

Independent variables	Dependent variables					
	R0 resection		Complication		30-day mortality	
Reason for conversion	Coefficient	*p*-value	Coefficient	*p*-value	Coefficient	*p*-value
Bleeding	0.051	0.427	−0.064	0.381	0.005	0.591
Technical difficulties	−0.005	0.938	−0.02	0.735	−0.002	0.857
Oncological concerns	−0.078	0.228	0.059	0.274	−0.005	0.577
Iatrogenic injuries	0.077	0.466	−0.019	0.856	0.041	0.402

### Conversion to open and Clavien-Dindo ≥3 complications

Analysis of 4,711 patients from 34 studies showed that Clavien-Dindo ≥3 complications occurred in 18.7% (95% CI 15.0–22.3) (Figure 2b). Multivariable meta-regression analysis showed that conversion to open did not affect Clavien-Dindo ≥3 complications (coefficient: 0.129, *p*=0.609) (Table 1, Figure 3b). Subgroup analysis based on reasons for conversion showed that Clavien-Dindo ≥3 complications were not affected by conversion due to bleeding (coefficient: −0.064, *p*=0.381), conversion due to technical difficulties (coefficient: −0.020, *p*=0.735), conversion due to oncological concerns (coefficient: 0.059, *p*=0.274), and conversion due to iatrogenic injuries (coefficient: −0.019, *p*=0.856) (Table 2). Comparative meta-analysis of 303 patients from three studies showed no difference in Clavien-Dindo ≥3 complications between patients with and without open conversion (OR: 2.17, 95% CI 0.67–6.99, *p*=0.20) (Figure 4b). The GRADE certainty was moderate.

### Conversion to open and 30-day mortality

Analysis of 4,231 patients from 33 studies showed that 30-day mortality occurred in 1.2% (95% CI 0.9–1.5) (Figure 2c). Multivariable meta-regression analysis showed that conversion to open did not affect 30-day mortality (coefficient: −0.013, *p*=0.647) (Table 1, Figure 3c). Subgroup analysis based on reasons for conversion showed that 30-day mortality was not affected by conversion due to bleeding (coefficient: 0.005, *p*=0.591), conversion due to technical difficulties (coefficient: −0.002, *p*=0.857), conversion due to oncological concerns (coefficient: −0.005, *p*=0.577) and conversion due to iatrogenic injuries (coefficient: 0.041, *p*=0.402) (Table 2). Comparative meta-analysis of 551 patients from four studies showed no difference in 30-day mortality between patients with and without open conversion (RD: 0.02, 95% CI −0.04–0.07, *p*=0.57) (Figure 4c). The GRADE certainty was moderate.

### Sensitivity analyses

Leave-one-out analysis and separate analysis for studies with low overall risk of bias did not affect overall conclusions.

## Discussion

We conducted a meta-analysis with meta-regression to investigate the prognostic significance of unplanned conversion to open surgery during laparoscopic pancreaticoduodenectomy. Analysis of 6,108 patients from 44 studies via proportion and comparison meta-analysis models showed that conversion to open surgery did not affect R0 resection, Clavien-Dindo ≥3 complications and 30-day mortality in patients undergoing laparoscopic pancreaticoduodenectomy. The reasons for conversion did not affect the outcomes and the GRADE certainty of evidence was moderate.

This study is the first meta-analysis with a large sample size to comprehensively evaluate effect of conversion to open during laparoscopic pancreaticoduodenectomy on resection margins and complications. While the effect of conversion on R0 resection cannot be compared directly with previous reviews, effect of conversion on complications can be compared with the findings of Karunakaran *et al*,^54^ who conducted a systematic review of 6 studies including patients undergoing any types of minimally invasive pancreaticoduodenectomy. Unlike the findings of the current study, Karunakaran *et al* concluded that conversion to open increased mortality and morbidity after minimally invasive pancreaticoduodenectomy.^54^ The difference between the findings of the current study and the study by Karunakaran *et al* can be explained by several factors. First, the number of included studies in the current study is significantly higher than the study by Karunakaran *et al*.^54^ Second, the current study only included patients undergoing laparoscopic pancreaticoduodenectomy while the study by Karunakaran *et al* combined both laparoscopic and robotic procedures, which may affect the reported complications rates.^54^ Therefore, the advantages of the current study in comparison with the study by Karunakaran *et al* may include a significantly larger number of included studies,^54^ inclusion of only laparoscopic cases, conducting meta-regression analyses based on the reasons for unplanned conversion and, more importantly, evaluating the effect of conversion on resection margins.

The findings of current study regarding the effect of unplanned conversion to open on the outcomes are novel. The reasons for conversion in order of frequency included technical difficulties, oncological concerns, bleeding and iatrogenic injuries; none affected R0 resection and complications. Although conversion to open did not improve the outcomes, it did not worsen the outcomes. This is a crucial finding as it is very important to recognise that avoiding conversion to open when indicated does increase the risk of postoperative morbidity and mortality. This benefit of conversion to open cannot be demonstrated in clinical research due to confounding by indication; conversion to open is ethically and clinically indicated to avoid negative outcome; hence, negative outcome of not converting can never be demonstrated. Therefore, conversion to open should not be considered as a failure and the operating surgeon should not hesitate to convert if there is concern that complete and safe resection cannot be achieved via minimally invasive approach.

The current study has some strengths and limitations. The sample size was large, reducing the risk of type 2 error; this can be reflected by relatively narrow CIs for most of the reported outcomes. The sensitivity analyses showed consistency of the results. The main limitation of the current study is the fact that none of the available studies in the literature is powered based on conversion to open surgery and only four studies compared patients with and without conversion directly; therefore, only four studies were included in the direct comparison meta-analysis model. Nevertheless, we constructed a meta-regression model as a well-recognised alternative approach in evidence synthesis to evaluate conversion to open as a prognostic factor. The statistical between-study heterogeneity was high, and this may be explained by differences among the included studies in terms of the centre volume, surgeons’ experience, and baseline characteristics of the included patients; nevertheless, we conducted sensitivity analyses and also downgraded the certainty of the evidence to compensate for this limitation.

## Conclusions

Unplanned conversion to open surgery, regardless of the reason for conversion, may not affect resection margins and complications in laparoscopic pancreaticoduodenectomy (moderate certainty). Conversion during laparoscopic pancreaticoduodenectomy should not be seen as a failure because it has no negative impact on outcomes; however, not converting when indicated will undoubtedly have a negative impact.

## Declarations

The study did not receive any funding and the authors declared no conflicts of interest. The corresponding author (Shahab H) will provide any additional data related to this study on reasonable request. Patient consent and approval from Research Ethics Committees were not required in this study because the study design was a meta-analysis which did not have direct involvement of patients and used nonidentifiable data.
